# Monolithic integration of room-temperature multifunctional BaTiO_3_-CoFe_2_O_4_ epitaxial heterostructures on Si(001)

**DOI:** 10.1038/srep31870

**Published:** 2016-08-23

**Authors:** Mateusz Scigaj, Nico Dix, Jaume Gázquez, María Varela, Ignasi Fina, Neus Domingo, Gervasi Herranz, Vassil Skumryev, Josep Fontcuberta, Florencio Sánchez

**Affiliations:** 1 Institut de Ciència de Materials de Barcelona (ICMAB-CSIC), Campus UAB, Bellaterra 08193, Barcelona, Spain; 2 Dep. de Fisica, Universitat Autònoma de Barcelona, Campus UAB, Bellaterra 08193, Barcelona, Spain; 3 Dep. Física Aplicada III & Instituto Pluridisciplinar, Universidad Complutense de Madrid, Madrid 28040, Spain; 4 Catalan Institute of Nanoscience and Nanotechnology (ICN2), CSIC and The Barcelona Institute of Science and Technology, Campus UAB, Bellaterra, 08193 Barcelona, Spain; 5 Institució Catalana de Recerca i Estudis Avançats (ICREA), Barcelona, Spain

## Abstract

The multifunctional (ferromagnetic and ferroelectric) response at room temperature that is elusive in single phase multiferroic materials can be achieved in a proper combination of ferroelectric perovskites and ferrimagnetic spinel oxides in horizontal heterostructures. In this work, lead-free CoFe_2_O_4_/BaTiO_3_ bilayers are integrated with Si(001) using LaNiO_3_/CeO_2_/YSZ as a tri-layer buffer. They present structural and functional properties close to those achieved on perovskite substrates: the bilayers are fully epitaxial with extremely flat surface, and exhibit robust ferromagnetism and ferroelectricity at room temperature.

Crystalline oxides, with a rich variety of functional properties, are major candidates for radical improvement of existing microelectronic devices or to develop new ones. For example, while the two switchable states of remnant magnetization in ferromagnetic (FM) oxides allow storing information, the switchable remnant polarization in ferroelectric (FE) oxides can be used for non-volatile electric-based memory devices. FM and FE oxides have found some niche applications in magnetic random access memories and in ferroelectric random access memories. However, the difficult monolithic integration of functional oxides with silicon^1^,^2^ is a bottleneck towards applications. Many crystalline oxides can be grown on Si wafers by using buffer layers^3^, but their structural characteristics and functional properties are generally poor in comparison with films on perovskite substrates. Promisingly, there has been significant progress in the monolithic integration of lead-free ferroelectrics on Si(001), including *a*-oriented BaTiO_3_ (BTO) films with high electrooptical responses for photonic devices^4^,^5^. The growth of *c*-oriented BTO films is more difficult on silicon wafers than on oxide substrates due to the tensile stress caused by the thermal mismatch (Si has much smaller thermal expansion coefficient than BTO). Buffer layers causing compressive epitaxial stress have permitted *c*-oriented BTO films with high ferroelectric polarization^6^, or ultrathin BTO layers for FE tunnel junctions^7^,^8^. Also, high quality epitaxial growth of vertical nanocomposites formed by the FE perovskite BiFeO_3_ and the FM insulator spinel CoFe_2_O_4_ (CFO) on silicon has been reported^9^. The vertical nanocomposites are an artificial multiferroic system with coexistence of FE and FM at room temperature. Other class of artificial multiferroics, more convenient for device fabrication, are horizontal heterostructures with FE and FM films^10^,^11^,^12^. However, perovskites and spinels are highly dissimilar, which hinders epitaxial growth when they are combined in horizontal heterostructures. This additional difficulty makes the integration of epitaxial horizontal bilayers of multiferroic oxides with Si, with structural and functional properties close to those of bilayers on perovskite substrates, extremely challenging.

Although epitaxial growth has been reported^13^, multiferroic oxide thin film bilayers on Si are generally polycrystalline and present high surface roughness^14^,^15^,^16^,^17^. Most of the reported multiferroic bilayers include PbZr_x_Ti_1−x_O_3_ (PZT) as FE oxide. However, Pb-free ferroelectrics are preferable, and the recent progress in the epitaxial integration of BTO films on Si(001) paves the way towards this goal^4^,^6^,^18^,^19^. Building on these advances, here we report on epitaxial horizontal top-CFO/bottom-BTO heterostructures on Si(001) buffered with LaNiO_3_ (LNO), CeO_2_ and yttria-stabilized zirconia (YSZ). The CFO/BTO bilayers here reported display a combination of structural and functional properties until now elusive in either Pb-free or PZT-based multiferroic bilayers: high structural quality together with robust multiferroicity at room temperature, with high saturation magnetization above 200 emu/cm^3^, remnant polarization around 20 μC/cm^2^, and low electrical leakage and high ferroelectric endurance.

## Results

### Samples growth and structural characterization

**Figure 1:**
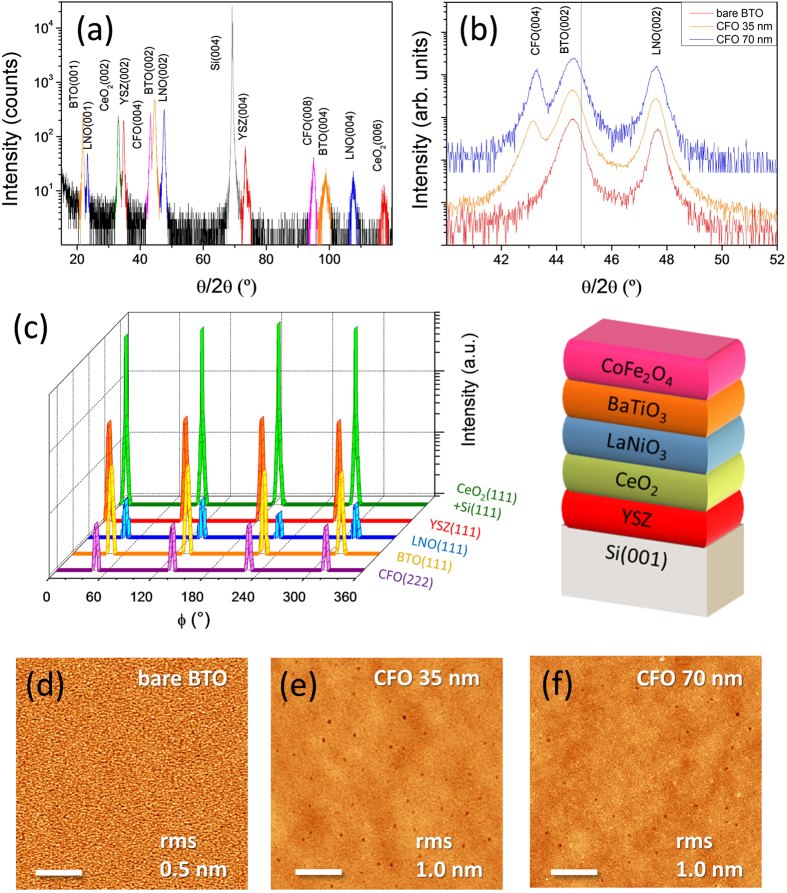
Epitaxial growth of flat complex oxide heterostructures on silicon. **(a)** XRD θ–2θ scan around symmetrical reflections of the t_CFO_ = 70 nm sample. **(b)** Zoom of the symmetrical θ–2θ scan around the CFO(004), BTO(002) and LNO(002) reflections of t_CFO_ = 70 and 35 nm samples and the sample with no CFO layer (t_CFO_ = 0 nm). For clarity the scans are shifted vertically. The vertical dashed line marks the position of the BTO(002) reflection in bulk BTO. **(c)** XRD ϕ-scans around asymmetrical reflections of the t_CFO_ = 70 nm sample. A sketch of the heterostructure is at the right. Topographic AFM images of t_CFO_ = 0 **(d)**, 35 **(e)** and 70 nm **(f)** samples. The scale bar is 1 μm and the rms roughness of each sample is indicated at the right of the corresponding image.

The strong chemical reactivity prevents epitaxy of CFO/BTO when they are directly deposited on bare Si(001) and therefore a buffer layer is required. We have selected YSZ, one of the few oxides that can be grown epitaxially directly on Si(001), even without removing the native oxide^20^. However, there is a high lattice mismatch of around 9% between YSZ and BTO, and additional oxide layers are necessary for a gradual matching of the crystal lattices. For this purpose, we deposited sequentially CeO_2_ and LNO layers on YSZ before growing the ferroelectric BTO. The multilayer buffer accommodates progressively the compressive stress and, overcoming the tensile stress caused by the thermal expansion mismatch between BTO and Si, favors the *c*-orientation of BTO^6^. A series of CFO/BTO bilayers with different thickness of CFO (t_CFO_) was fabricated. The X-ray diffractometry (XRD) θ–2θ scan around symmetrical reflections (Fig. 1a), measured in the CFO/BTO/LNO/CeO_2_/YSZ/Si(001) sample with the thickest CFO (t_CFO_ = 70 nm), shows (00l) reflections from the substrate and the five layers in the heterostructure, with absence of other orientations and secondary phases. Figure 1b shows a zoom around the CFO(004), BTO(002) and LNO(002) reflections for this sample and t_CFO_ = 35 and 0 nm samples. The intensity of the CFO(004) peak changes accordingly with the thickness of CFO in each sample. BTO(002) peaks are at lower angles than in bulk (the vertical line marks the position of the (002) reflection for bulk BTO), signaling that BTO is *c*-oriented with an expanded out-of-plane lattice parameter of around 4.06 Å. The heterostructure is epitaxial as confirmed by XRD ϕ-scans around asymmetrical reflections of the layers (Fig. 1c), with cube-on-cube growth of CeO_2_ and YSZ and 45° in-plane rotation of LNO, BTO and CFO respect to the Si(001) substrate.

Even though the heterostructure includes five layers (see a sketch in Fig. 1c) with high structural dissimilarity, the samples present a very flat surface. Topographic atomic force microscopy (AFM) scans, 5 μm × 5 μm in size, corresponding to t_CFO_ = 0, 35 and 70 nm samples are presented in Fig. 1d–f. The bare BTO film (t_CFO_ = 0 nm sample) exhibits small islands, with lateral dimensions of a few tens of nm and overall rms roughness as low as 5 Å. The growth of flat spinel (001) oriented CFO films is challenging since they tend to be rough by forming {111} facetted pyramidal islands, due to its lower surface energy. We recently showed^12^ that flat spinel films can be grown on (001)-oriented perovskite substrates under proper kinetic limitations. That is the reason why, CFO films on BTO/LNO/CeO_2_/YSZ/Si(001) have been grown at relatively low substrate temperature (500 °C) and high deposition rate (laser frequency was 5 Hz). CFO films, as thick as 35 nm (Fig. 1e) and 70 nm (Fig. 1f) deposited using these parameters, are slightly rougher (rms = 10 Å) than the bottom BTO layers, with presence of some small regions a few nm deeper, likely due to coalescence of nanometric pyramidal islands^12^.

**Figure 2:**
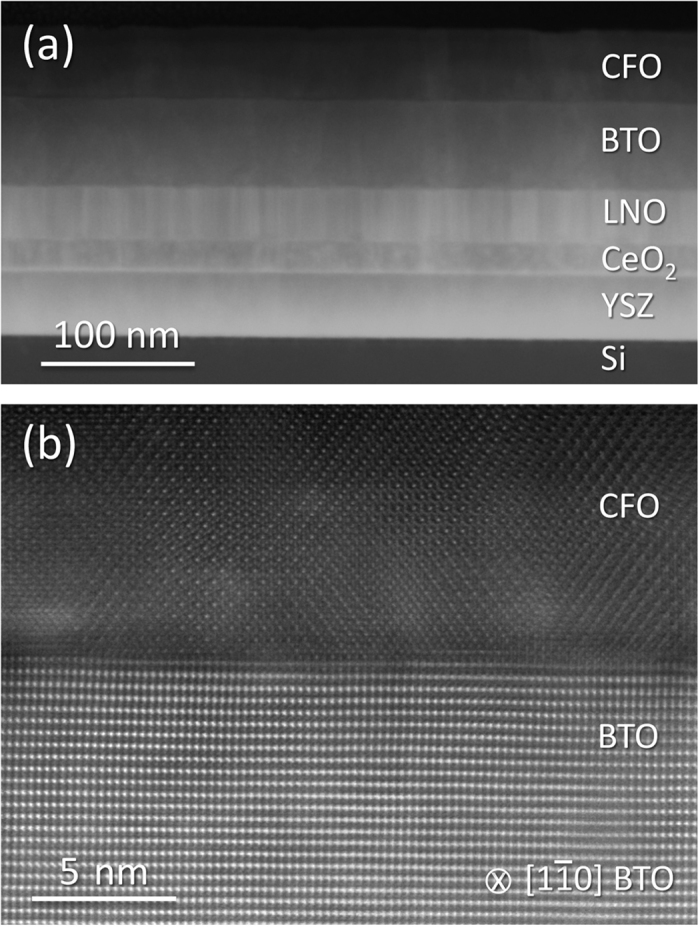
Epitaxial oxide heterostructures on silicon with sharp interfaces. **(a)** Low magnification STEM high angle annular dark field image along the Si[100] zone axis. All the five films of the multilayer are continuous over long lateral lengths. No secondary phases are observed. **(b)** High resolution image of the interface between the perovskite BTO and the spinel CFO along the BTO[110] zone axis.

Figure 2a shows a scanning transmission electron microscopy (STEM) cross section image recorded by a high-angle annular dark field (HAADF) detector of a heterostructure with a CFO layer around 50 nm thick. The image manifests the quality of the sample, with uniform thickness of all the layers over all the imaged lateral length. Secondary phases are not observed either within the different layers or at any of the interfaces between them. A thin interfacial SiO_x_ layer (a few nm thick; not visible in the low magnification image) is present between the Si(001) substrate and the YSZ buffer layer. The high-resolution HAADF image of the CFO/BTO interface in Fig. 2b confirms the cube-on-cube epitaxial relationship between CFO and BTO. In the HAADF imaging mode the contrast scales with the square of the atomic number (Z) of the atoms columns and therefore the BTO layer, containing high-Z Ba, is brighter than the CFO layer. It allows identifying a sharp interface between the CFO and BTO layers.

### Ferroelectric properties

**Figure 3:**
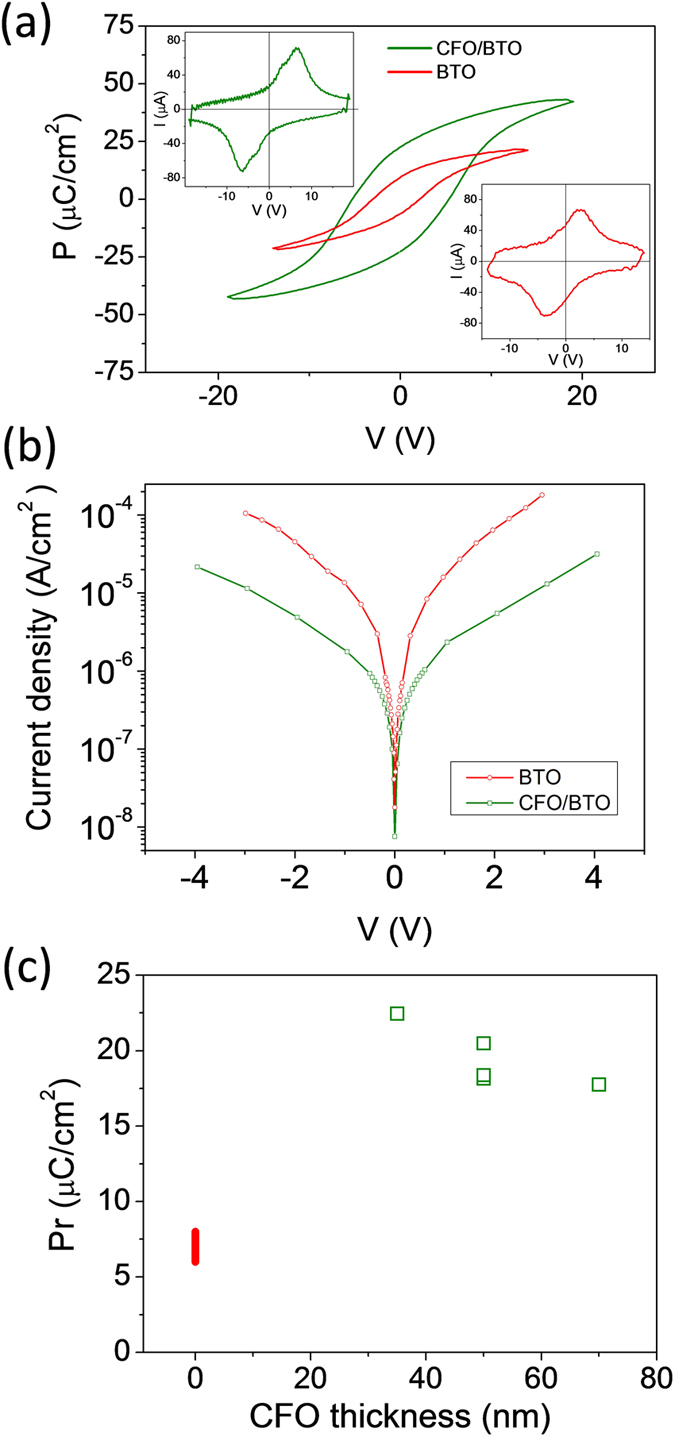
Electrical characterization of CFO/BTO bilayers. **(a)** Polarization – voltage loops of t_CFO_ = 35 nm CFO/BTO bilayer and the corresponding BTO single film (t_CFO_ = 0 nm). **(b)** Leakage current density versus voltage curves corresponding to t_CFO_ = 0 and 50 nm samples. **(c)** Remnant polarization versus CFO thickness (note that three different t_CFO_ = 50 nm samples were fabricated). The vertical bar in t_CFO_ = 0 nm indicates the range of polarization values measured in the series of single BTO films on LNO/CeO_2_/YSZ/Si(001) reported in ref. 6.

We show in Fig. 3a the P(V) loops of the CFO/BTO bilayer with t_CFO_ = 35 nm (green line) and the reference bare BTO film (red line). The corresponding current - voltage curves in the top left and bottom right insets, respectively, show clear ferroelectric switching peaks. The coercive voltage in the CFO/BTO bilayer (around 7.5 V) is higher than in the bare BTO film (around 3V), as expected considering the presence of the insulating CFO layer that reduces the effective electric field in the BTO layer (see Supplementary Information). Consistent with the insulating behavior of CFO, the measured leakage current across the Pt/CFO/BTO/LNO heterostructure is lower than across the Pt/BTO/LNO reference heterostructure, as it is shown in Fig. 3b. For instance, for an applied voltage of 2V the current density for the single BTO is around 5 × 10^−5 ^A/cm^2^, whereas its value is 5 × 10^−6 ^A/cm^2 ^for the CFO/BTO bilayer. These values are comparable to the leakage measured in thicker ferroelectric PZT^21^ and BiFeO_3_^22^ single layers and PZT/spinel bilayers^14^ on Si(001). The high ferroelectric polarization of the CFO/BTO bilayer (Fig. 3a) is an outstanding result. In particular, we emphasize: i) the observation of a ferroelectric polarization in a bilayer system which includes a thick dielectric layer (t_CFO_ = 35 nm), and ii) the higher remnant polarization of the bilayer (≈23 μC/cm^2^) compared with the bare BTO film (below 7 μC/cm^2^). Notice that result (i) is remarkable since in presence of insulating top CFO layer, the depolarizing field acting on BTO should preclude the establishment of a monodomain polar state along the out-of-plane direction. An ultrathin CFO layer is likely to be polarized according to the polarization state of BTO and, therefore, compensation charges provided by the Pt electrode should build at the top of the CFO surface. However, this mechanism is only energetically available for ultrathin dielectric layers^23^. A more likely alternative scenario would be that BTO (or CFO) free-charges migrate to the interface to compensate the net polarization at BTO interface. The second remarkable result is that the large remnant polarization (≈23 μC/cm^2^) of the bilayer is much higher than that of the reference bare BTO film (below 7 μC/cm^2^), being the BTO strain similar in both samples, as shown in Fig. 1b. The high polarization, with Pr ≈ 20 μC/cm^2^, has been measured in the five bilayers investigated (Fig. 3b). The red vertical bar for t_CFO_ = 0 nm in the graph indicates the range of remnant polarization (6–8 μC/cm^2^) that we reported^6^ for a series of single BTO films on LNO/CeO_2_/YSZ/Si(001). Therefore the remnant polarization of the bilayers is enhanced by about a factor ~3 with respect to the equivalent bare BTO films. Although the origin of this enhancement is unclear, we note that the electrical boundary conditions in bare and CFO capped BTO layer are different from each other when the samples are cooled down after deposition through a paraelectric-ferroelectric phase transition. In particular, in the case of the bare BTO layer, its surface is exposed to the vacuum. This results in the largest possible depolarizing field^24^,^25^, which can favour the presence of compensating domains. The presence of these compensating domains degrades the switchability (because they promote the pinning of the polarization) and favour low retention (because they would generate stray fields). In the case of CFO/BTO, CFO is a bad insulator and it might provide compensating free-charges.

**Figure 4:**
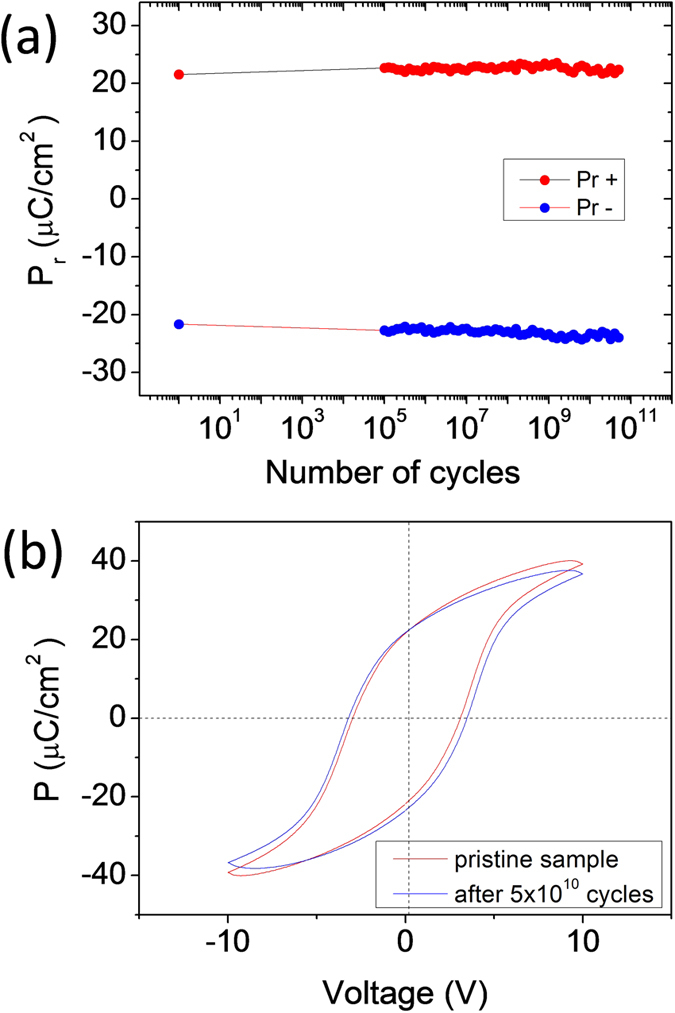
Ferroelectric endurance. **(a)** Polarization fatigue as a function of the number of cycles measured at room temperature in a t_CFO_ = 50 nm sample. **(b)** Polarization – voltage loops of the pristine sample and after 5 × 10^10^ cycles.

Further ferroelectric characterization shows that CFO/BTO bilayers exhibit excellent endurance against ferroelectric fatigue. The remnant polarization of a t_CFO_ = 50 nm sample as a function of the number of switching cycles (up to 5 × 10^10^ pulses) is plotted in Fig. 4a. It can be inferred that the remnant polarization remains around 22 μC/cm^2^ during the fatigue test. The P-V loops of the pristine sample and after 5 × 10^10^ cycles (Fig. 4b) do not show remarkable differences.

**Figure 5:**
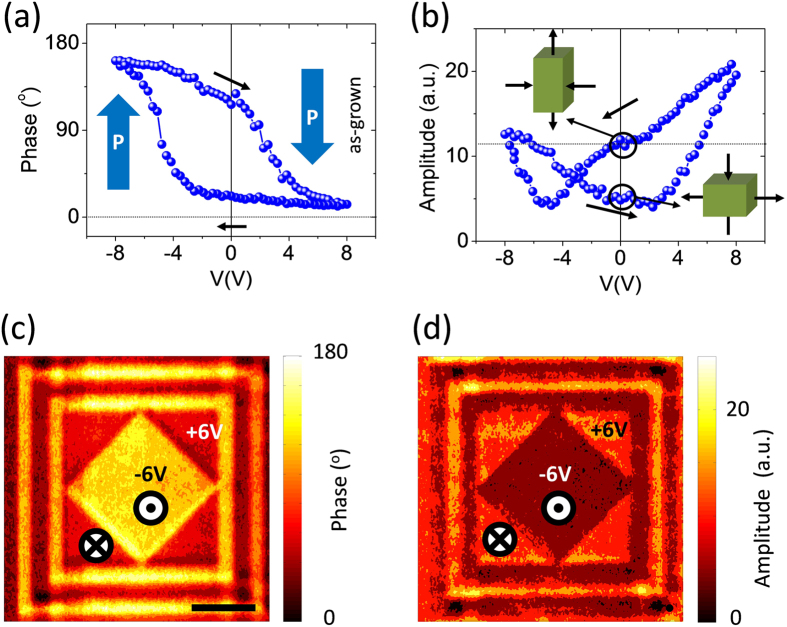
Piezoresponse force microscopy characterization. Piezoresponse phase **(a)** and piezoresponse amplitude **(b)** versus applied voltage loops, measured with an AC driving voltage Vac = 1 V. Piezoresponse phase **(c)** and piezoresponse amplitude **(d)** images recorded at Vac = 1 V, after poling with +6 V and −6 V. Scale bar corresponds to 4 μm.

The piezoelectric response of the bilayers has been studied by piezoresponse force microscopy (PFM). Typical PFM hysteresis loops, i.e. phase (Φ) and amplitude (proportional to the piezoelectric coefficient d_33_) versus applied voltage (V) are shown in Fig. 5(a,b). In Fig. 5a, 180° phase contrast upon cycling V demonstrates the piezoelectric nature of the characterized heterostructure. Sizeable imprint field shifting the whole loop towards the left is observed, most probably due to the asymmetry of the CFO/BTO heterostructure plus the asymmetric work-functions of top and bottom electrodes (the conductive tip of the cantilever and the LNO conductive oxide). The imprint field corresponds to an electric field towards the LNO bottom electrode, which leads to an as-grown state where polarization is pointing down (P_down_). Equivalent, in Fig. 5b, it can be observed the typical ferroelectric butterfly hysteresis loop for the amplitude response. The imprint field is also visible. In the case of the piezoamplitude response the imprint field is responsible for the two bi-stable strain states observed at zero voltage. As it can be inferred from Fig. 5b, for the as-grown and P_down_ states, the strain state corresponds to a more extended out-of-plain parameter than for the P_up_ state. Note also in Fig. 5(a,b) that the coercive voltage obtained (≈6 V) is similar to the value obtained using macroscopic contacts (Fig. 4c). The capability to write up and down ferroelectric domains by application of external bias voltage induced by the AFM tip is summarized by Fig. 5(c,d). The phase of the piezoresponse signal (with near 180^o^ contrast) after poling the sample with +6 (dark) and −6 V (brigth) is displayed in Fig. 5c. In Fig. 5d, the equivalent measurement for the amplitude is shown, where the contrast in the image is due to the mentioned imprint field, which lead to two remanent values for the amplitude.

### Magnetic properties

**Figure 6:**
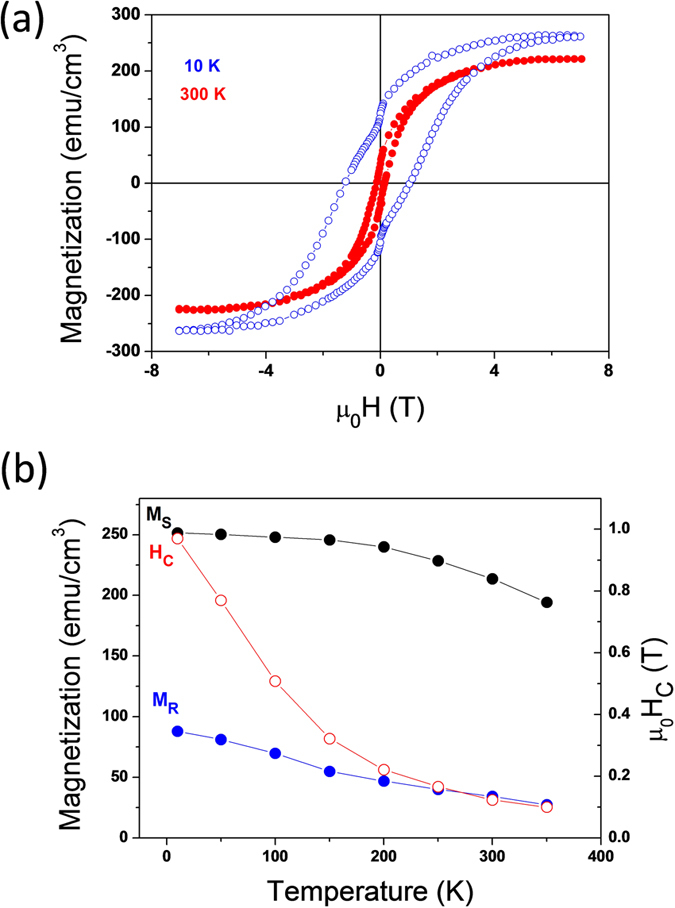
Magnetic properties. **(a)** Magnetization loops (t_CFO_ = 70 nm sample) measured at 10 (empty blue circles) and 300 K (solid red circles) with the field applied in-plane along Si[110]. **(b)** Temperature dependence of the saturation and remnant magnetization (left axis) and the coercive field (right axis).

Magnetization loops were measured on the t_CFO_ = 70 nm sample with the field applied along the CFO[100] in-plane direction at several temperatures within the 10–350 K range. Selected hysteresis loops, measured at 10 and 300 K, are plotted in Fig. 6a. The presence of a double step in the M(H) loops of thin films of spinel oxides^26^,^27^,^28^,^29^,^30^,^31^ and other ferrimagnetic oxide as ε-Fe_2_O_3_^32^ as well as ceramic samples^33^ is a common experimental observation. The saturation magnetization M_s_, about 250 emu/cm^3^ at 10 K, is lower than in bulk CFO as usually observed in CFO films deposited on perovskite oxide surfaces^26^,^27^,^28^,^30^. M_s_ decreases only slightly with temperature (Fig. 6b) and remains above 200 emu/cm^3^ up to 350 K (the highest temperature in our measurements), suggesting that the Curie temperature is well above room temperature as in the bulk material^34^. In Fig. 6b the coercive field is also plotted as a function of the temperature. It decreases from about 1.0 T at 10 K to 0.1 T at 350 K, reflecting the change in the magnetic anisotropy. Accordingly, the remnant magnetization to the saturation magnetization ratio is about 0.46 and 0.17 at 10 K and room temperature, respectively.

### Magnetoelectric characterization

**Figure 7:**
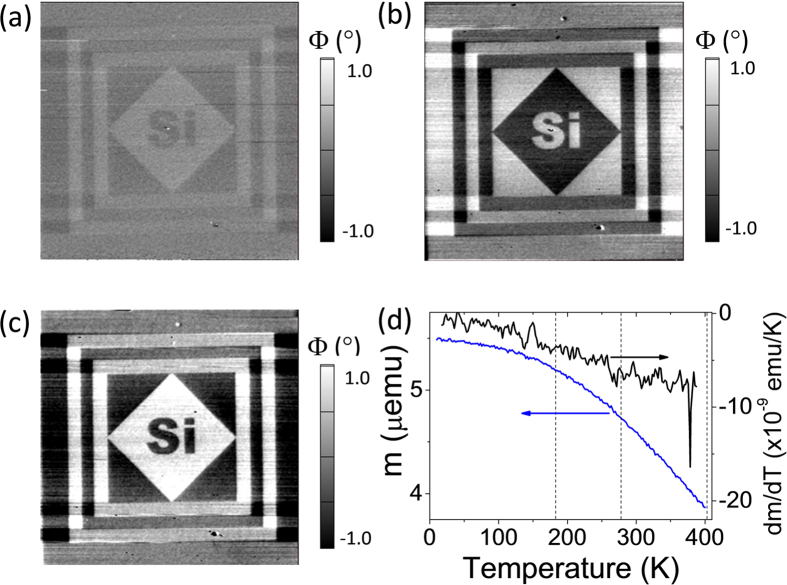
Magnetoelectric coupling measurements. **(a)** Magnetic force microscopy phase image after poling at −6 V (bright) and +6 V (dark). **(b)** and **(c)** Magnetic force microscopy phase image recorded biasing the tip with −2 V and +2 V, respectively. In (**a–c**) the figure size is 40 μm by 40 μm. **(d)** Temperature dependence of the magnetization (measured by SQUID) and its derivative (right-axis).

The combination of ferroelectric and ferromagnetic films in multiferroic epitaxial heterostructures could permit magnetoelectric coupling. In the present case, where the magnetic layer is insulating, an electric field-effect arising from the polarization acting on the CFO can be neglected. Therefore, only a direct coupling mediated by the elastic interaction between the films (magnetoelastic coupling) could eventually exist. However, in epitaxial films, the layers are clamped to the substrate, and the elastic coupling is expected to depend largely on the materials and their microstructure^35^. Indeed, in bilayers formed by top CFO on BTO or PZT films, a strong dependence of the magnetoelectric coupling on crystal orientation, relative thicknesses, buffer layers used, and direction of the applied magnetic field has been observed^36^,^37^,^38^. The magnetoelectric coupling coefficients have been found ranging from negligible up to about 100 mV/cm∙Oe. We have investigated the eventual existence of converse magnetoelectric effect in our samples by magnetic force microscopy (MFM) and SQUID measurements. With this purpose, we first poled some regions of the CFO/BTO bilayer by −6 V (bright) and +6 V (dark). Next, MFM phase contrast images were recorded (Fig. 7a)). The clear contrast observed in the MFM images in Fig. 7 replicates the written poled pattern. At first sight the observed contrast could be interpreted as of magnetic origin, and accordingly it would be a signature of magnetoelectric coupling. However the contrast in the MFM image of Fig. 7a, could also result from the electrostatic interaction between the metallic and magnetic tip used in the MFM scans and possible surface charges at the written domains. To discriminate between these two scenarios, two additional MFM images (Fig. 7b,c) of the same region were sequentially recorded while biasing the magnetic tip with −2 and +2 V, respectively. The increase of contrast in Fig. 7b and the contrast reversal in Fig. 7c upon changing the sign of the tip voltage indicate that the observe contrast is dominated by electrostatic charges, and thus the magnetoelectric effect, if any, remains imperceptible. In other experiment (not shown here) we explored the existence of direct magnetoelectric coupling by measuring ferroelectric loops under a magnetic field applied in-plane, with intensity values up to 1 T. The polarization did not show changes with the applied magnetic field. At a glance, these results contrast with some reportedly large magnetoelectric coupling coefficients (about 100 mV/cm∙Oe) in CFO/BTO bilayers on perovskite substrates^38^. However, it should be noted that these last values were obtained from measurements where a time-dependent H_ac_ field was superimposed to a H_dc_ field and the corresponding electromotive force is recorded. Therefore, a dynamic magnetoelectric coupling coefficient was determined there^36^,^37^,^38^ which is radically different from the static quasi-equilibrium response determined here. Understanding the microscopic mechanism leading to these different responses and consequently to different magnetoelectric coupling coefficients, would certainly be of interest but it is beyond the scope of this paper.

We also investigated the possible existence of a converse magnetoelectric coupling by using an indirect method. BTO is tetragonal at room temperature, but it becomes first orthorhombic and next rhombohedral when lowering the temperatures, and cubic when increasing the temperature above T_c_ ~ 400 K. These structural changes are accompanied by rotation of the polarization axes and subsequent formation of domains. These changes should propagate into the magnetic layer producing noticeable changes of their properties, as clearly observed in magnetic layers grown on BTO crystals^39^. Following this approach, the temperature dependence of the magnetization of CFO at H = 30 Oe applied in-plane was measured by SQUID (Fig. 7d) aiming to detect magnetization changes at temperatures around the phase transitions (the vertical dashed lines mark the transitions in bulk BTO). However, anomalies were not appreciated, even in the derivative data (right-axis in Fig. 7d). This is in contrast with the magnetization changes reported for soft ferromagnetic layers grown on BTO single crystals^39^, and suggests that in the present case, where the BTO thin films is epitaxially grown on a passive substrate (Si), the epitaxial clamping blocks drastically the phase transformation in BTO^40^ and consequently the magnetic properties of CFO are not modified.

## Discussion

In summary, CFO/BTO bilayers have been monolithically integrated on Si(001) with a LNO/CeO_2_/YSZ buffer. The samples are very flat and epitaxial, presenting *c*-oriented BTO with an expanded out-of-plane lattice parameter. The CFO/BTO bilayers show multifunctional properties at room temperature, with high magnetization and excellent ferroelectric properties. These artificial multiferroics are thus potential candidates for non-volatile four states multiferroic monolithic memories.

To end, we note that our work can be relevant for other memory devices. First, for FE-field effect transistors (FE-FETs)^41^, which are currently limited by insufficient retention time and endurance. They are usually based on FE/insulator heterostructures on silicon, and our observation of robust ferroelectricity in CoFe_2_O_4_/FE bilayers suggests that a proper selection of the insulator can permit an improvement in the performance of FE-FETs memories. Second, the two bi-stable strain states in the piezoresponse of the CFO/BTO bilayers is of potential interest as on alternative approach in piezotronic (stress field controlled) memories^42^. In patterned CFO/BTO (or alternatively soft FM metal/BTO) nanostructures the deformation state of the bottom piezo BTO could control the magnetic anisotropy of the top FM layer, defining two non-volatile magnetization states.

## Methods

### Thin films growth

The epitaxial heterostructures were grown on as-received Si(001) by pulsed laser deposition using a KrF excimer laser. A multitarget stage with ceramic YSZ, CeO_2_, LNO, BTO and CFO was used to fabricate CFO/BTO/LNO/CeO_2_/YSZ/Si(001) samples in a single process. In a series of samples the thickness of CFO (t_CFO_) was varied from 35 to 70 nm, with fixed thickness of around 40, 20, 35 and 55 nm for YSZ, CeO_2_, LNO, and BTO, respectively. Additional experimental details are reported elsewhere^6^,^12^. Square Pt top contacts (20  nm thick, 60  μm wide) were deposited ex-situ through shadow masks.

### Structural characterization

Epitaxial relationships and lattice parameters were determined by XRD using Cu Kα radiation. Topographic images of the heterostructures surface were recorded by AFM in dynamic mode. A heterostructure was observed in cross sectional view by a JEOL ARM 200CF STEM with a cold field emission source operated at 200 kV and equipped with a CEOS aberration corrector.

### Electrical characterization

Ferroelectric polarization loops, and leakage current density measurements were performed at room temperature contacting two top Pt-electrodes and with the conducting LNO buffer layer as common bottom electrode. In this configuration two CFO/BTO capacitors are measured in series. The measurements were performed at room temperature using a TFAnalyser2000 platform (aixACCT Systems). Ferroelectric loops were obtained measuring the current while sweeping the electric field at constant rate with frequencies of 4 kHz^43^. The polarization (P) was obtained after current integration through time and normalized to the electrode area. Loops were compensated by the dielectric leakage current compensation (DLCC) technique^43^,^44^ and series resistance contribution removed in order to accurately determine the ferroelectric parameters in presence of low leakage (see Supplementary Information). Leakage current at a specific voltage was measured with an integration time of 3 s, and the leakage curves were obtained averaging I-V curves increasing and decreasing voltage. Fatigue analysis was performed determining the remnant polarization from the measurement at room temperature of the P-V loops (contacting top Pt and bottom LNO electrodes) after cycling n-times the sample with 10 μs voltage pulses of 10 V (long enough to fully polarize the sample) using both polarities.

### Piezoresponse force microscopy

PFM measurements were performed with a MFP-3D Asylum Research microscope. MikroMasch silicon cantilevers with Pt coating (ANSCM-PT) were used. To achieve better sensitivity, the dual AC resonance tracking (DART) method was employed^45^,^46^. PFM voltage hysteresis loops were always performed at remanence, using a dwell time of 100 ms.

### Magnetic characterization

Magnetization loops were measured in the 10–350 K range by superconducting quantum interference device (SQUID) with the magnetic field applied in plane along Si[110]. Magnetization dependence on temperature has been measured using the same platform and conditions after demagnetizing the sample to 5.5 μemu at low temperature.

### Magnetoelectric coupling characterization

MFM measurements have been performed using the same AFM platform than in the PFM measurements. In MFM measurements the phase shift near the cantilever resonance is tracked while keeping the cantilever at a constant distance (35 nm) from the surface. The cantilever (model MFM Hc from NT-MDT) had been magnetized with a magnet before imaging.

## Additional Information

**How to cite this article**: Scigaj, M. *et al*. Monolithic integration of room-temperature multifunctional BaTiO_3_-CoFe_2_O_4_ epitaxial heterostructures on Si(001). *Sci. Rep.* **6**, 31870; doi: 10.1038/srep31870 (2016).

## Supplementary Material

Supplementary Information
