# Supplementary material for: Monolithic integration of room-temperature multifunctional BaTiO3-CoFe2O4 epitaxial heterostructures on Si(001)
Source: Sci Rep. 2016 Aug 23;6:31870. doi: 10.1038/srep31870 (PMC4994007; doi:10.1038/srep31870)

Supplementary Information

Monolithic integration of room-temperature multifunctional $BaTiO_3$-$CoFe_2O_4$ epitaxial heterostructures on Si(001)

Mateusz Scigaj,[1,2] Nico Dix,[1] Jaume Gázquez,[1] María Varela,[3,4] Ignasi Fina,[1,5] Neus Domingo[5], Gervasi Herranz,[1] Vassil Skumryev,[2,6] Josep Fontcuberta,[1] and Florencio Sánchez[1]

[1] Institut de Ciència de Materials de Barcelona (ICMAB-CSIC), Campus UAB, Bellaterra 08193, Barcelona, Spain

[2] Dep. de Fisica, Universitat Autònoma de Barcelona, Campus UAB, Bellaterra 08193, Barcelona, Spain

[3] Dep. Física Aplicada III & Instituto Pluridisciplinar, Universidad Complutense de Madrid, Madrid 28040, Spain,

[4] Materials Science and Technology Division, Oak Ridge National Laboratory, Oak Ridge, TN 37831, USA.

[5] Catalan Institute of Nanoscience and Nanotechnology (ICN2), CSIC and The Barcelona Institute of Science and Technology, Campus UAB, Bellaterra, 08193 Barcelona, Spain

[6] Institució Catalana de Recerca i Estudis Avançats (ICREA), Barcelona, Spain

fsanchez@icmab.es

Supplementary Note 1:

Our bilayer system can be roughly described as two series capacitors. The equation that describe two series capacitors are:

$$\begin{cases} Q = C_{BTO}V_{BTO} \\ Q = C_{CFO}V_{CFO} \\ 1/C_{total} = 1/C_{BTO} + 1/C_{CFO} \end{cases}$$

Where Q is the total charge stored in the system, C_{CFO} and C_{BTO} are the capacitances of each capacitor (equivalent to the CFO and BTO layers), the C_{total} is the total capacitance of the system and V_{CFO} and V_{BTO} are the equivalent voltage drop in each capacitor (equivalent to the CFO and BTO layers).

Using the relation for a planoparallel capacitor C=ε*A/t, it thus follows that:

$$V_{BTO} = \frac{\varepsilon_{CFO}/t_{CFO}}{\varepsilon_{CFO}/t_{CFO} + \varepsilon_{BTO}/t_{BTO}} V_{total}$$

and that ε_{CFO} = 14 [D. Gutiérrez et al, Physical Review B 86, 125309 (2012)], ε_{BTO} = 60 [Y.S. Kim et al., Applied Physics Letters 88, 072909 (2006)], t_{CFO} = 35 and t_{BTO} = 55 nm, it does follow that the measured coercive voltage 7.5 V correspond to a drop of voltage on the BTO layer of 2 V in agreement with the measured coercive voltage on the bare BTO sample.

Supplementary Figure 1: I-V loops recorded for both bare BTO (a) and CFO/BTO (b) samples, with and without DLCC compensation. It can be observed that in both samples DLCC only compensates the leakage contribution without producing a significant change in the amplitude/height of the current ferroelectric peak.

Only a slight increase of the current peak in Figure 1b for the CFO/BTO sample is observed due to in this sample the E_c variation on frequency is larger due to the presence of the CFO layer.

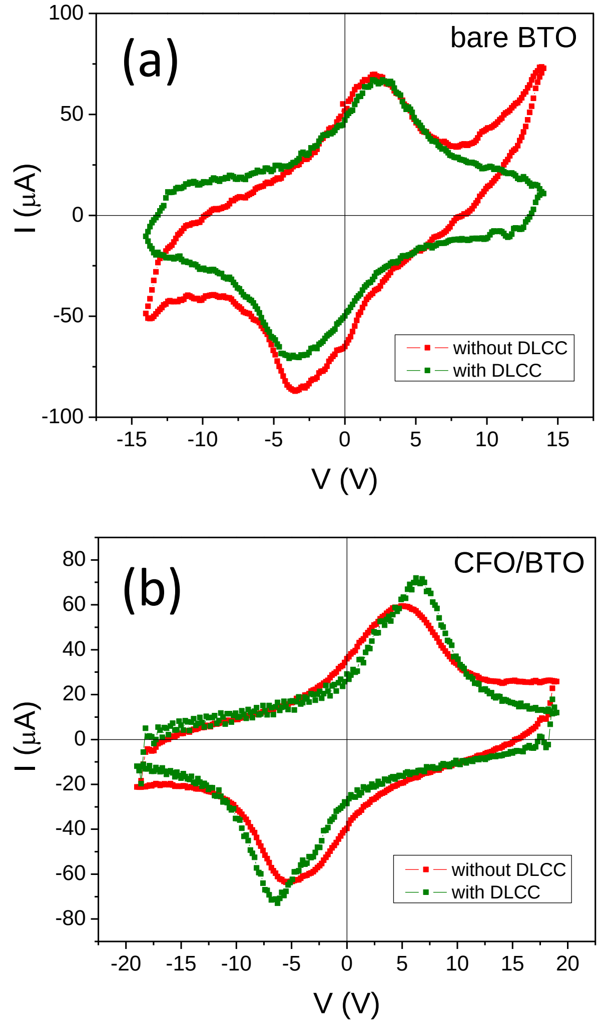

Supplement: Supplementary Information [file srep31870-s1.pdf]
